# A novel approach to conducting clinical trials in the community setting: utilizing patient-driven platforms and social media to drive web-based patient recruitment

**DOI:** 10.1186/s12874-020-00926-y

**Published:** 2020-03-13

**Authors:** Janelle Applequist, Cristina Burroughs, Artemio Ramirez, Peter A. Merkel, Marc E. Rothenberg, Bruce Trapnell, Robert J. Desnick, Mustafa Sahin, Jeffrey P. Krischer

**Affiliations:** 1 Zimmerman School of Advertising and Mass Communications, University of South Florida, 4202 E. Fowler Ave., CIS 1040, Tampa, FL 33620 USA; 2 Health Informatics Institute, University of South Florida, 3650 Spectrum Blvd., Suite 100, Tampa, FL 33612 USA; 3 Rheumatology Division, University of Pennsylvania, 3400 Spruce St., 5 White, Philadelphia, PA 19104 USA; 4 Department of Internal Medicine, University of Cincinnati College of Medicine, Medical Science Building, 231 Albert Sabin Way, P.O. Box 670515, Cincinnati, OH 45257-0515 USA; 5 Icahn School of Medicine at Mount Sinai, Icahn (East) Building, Floor 14, Room 14-34, 1425 Madison Ave, New York, NY 10029 USA; 6 F.M. Kirby Neurobiology Center, Boston Children’s Hospital, Center for Life Science, Room 14-073, 3 Blackfan Circle, Boston, MA 02115 USA

**Keywords:** Patient recruitment, Research recruitment, Clinical research, Web-based recruitment, Social media, Social media recruitment, Patient-centered research, Rare diseases, Social marketing theory

## Abstract

**Background:**

Participant recruitment for clinical research studies remains a significant challenge for researchers. Novel approaches to recruitment are necessary to ensure that populations are easier to reach. In the context of rare diseases, social media provides a unique opportunity for connecting with patient groups that have representatively lower diagnosis rates when compared with more common diseases or illness. We describe the implementation of designing a patient-centered approach to message design for the purposes of recruiting patients for clinical research studies for rare disease populations.

**Methods:**

Using an iterative research approach, we analyzed our previous experience of using web-based direct-to-patient recruitment methods to compare these online strategies with traditional center of excellence recruitment strategies. After choosing six research studies for inclusion in the previous study, in-depth, online interviews (*n* = 37) were conducted with patients represented in each disease category to develop and test recruitment message strategies for social media and a Web-based platform for patients to access study information and pre-screen. Finally, relationships were established with Patient Advocacy Groups representing each rare disease category to ensure further dissemination of recruitment materials via their own social media networks.

**Results:**

Guided by social marketing theory, we created and tested various recruitment message designs. Three key message concepts preferred by patients emerged: (1) infographic; (2) positive emotional messages; and (3) educational information for sharing. A base study website was designed and created based on data from patient interviews. This website includes the option for potential participants to pre-screen and determine their eligibility for the study.

**Conclusions:**

Study participants report wanting to be involved in the design and implementation of recruitment approaches for clinical research studies. The application of the aforementioned methods could aide in the evolution of clinical research practices for the recruitment of both rare and common diseases, where patient-centric approaches can help to create targeted messages designs that participants pre-test and support.

## Background

While medical research continues to provide great benefit to society in terms of advancing knowledge and innovation, achieving target rates for study recruitment and accrual remains a challenge. Inability to reach eligible patients for recruitment ultimately reduces the statistical power of studies, incurs economic costs, and may jeopardize funding [1–8]. In the United Kingdom, for example, research suggests that only 55–56% of randomized controlled trials achieve their target recruitment rate [9, 10].. In the conduct of many clinical research studies, especially for rare diseases, obtaining appropriate numbers of study participants at one single institution is often not feasible, creating a need for the organization of multi-center studies that require specially designed infrastructure. In addition to this model being costly, a major problem with the traditional research model is that the majority of potential study subjects are out of reach to the relatively few, geographically-limited clinical centers involved in the trial [11]. Rare disease populations present a particular challenge in terms of trial recruitment, with 32% of studies citing lack of patient accrual as the most common reason for trial non-completion [12].

The pharmaceutical industry has positioned itself prominently in the media landscape via its practice of direct-to-consumer advertising of prescription drugs, only allowed in the USA [13]. Such advertising provides the public with knowledge of available treatment options, while simultaneously attracting consumers to particular products [14, 15]. Although investigators are more limited in their recruitment budgets when compared to the pharmaceutical industry, the foundations of direct-to-consumer advertising feature methods that may prove useful in the context of research recruitment. Research has investigated such strategies used to increase recruitment and retention in clinical trials, finding that open trials (rather than blinded, placebo trials), telephone reminders to potential participants to respond to study invitations, information leaflets, and recruitment messages emphasizing scarcity are feasible approaches for improvement [16, 17]. In particular, the use of the Internet and social media platforms as recruitment tools provide areas for deeper exploration, as these sources provide cost-effective, and sometimes free, access to potential participants. Research has taken notice of this area in attempts to broaden participant reach in an attempt to overcome barriers to enrollment in clinical research studies [18–24].

Previous studies have investigated the use of paid advertisements via Facebook, a singular recruitment effort, but have found mixed results. Some studies have found that paid advertisements via Facebook are financially feasible, offer the ability to attract large numbers of individuals, and provide opportunities for connecting with individuals with specific health conditions [25–27]. Other studies, however, concluded that this type of recruitment may yield few participants, and if participants can be obtained, it is a costly process [28, 29].

Obtaining sufficient numbers of participants has continued to be a challenge for clinical research, but remains a significant battle in the context of rare diseases [30, 31]. Under the Rare Diseases Act of 2002, rare diseases are classified as those that affect < 200,000 persons in the United States [32]. Although each such disease may be rare, there are > 6000 rare diseases and the total number of people with at least one rare disease is large. Nonetheless, due to the low incidence of these individual diagnoses, the recruitment of sufficient numbers for research studies provides a great challenge.

The Rare Diseases Clinical Research Network (RDCRN) is an innovative international clinical research initiative of the Office of Rare Diseases Research (ORDR) and the National Center for Advancing Translational Sciences (NCATS) consisting of a network of 21 distinct clinical research consortia. The RDCRN coordinates research studies on more than 200 rare diseases. Centralized coordination is provided by the Data Management and Coordinating Center (DMCC) at the University of South Florida (USF). The DMCC houses all RDCRN data and organizes all protocol activity for more than 100 studies of the 21 rare disease consortia via in-house scalable and customizable electronic data capture systems.

Previously, the Vasculitis Clinical Research Consortium, a member of the RDCRN, has tested the use of web-based direct-to-patient recruitment methods in comparison with tradition multicenter recruitment strategies. In The Assessment of Prednisone in Remission (TAPIR) trial, online recruitment strategies via (Web-based and social media strategies) were tested for comparison with traditional center of excellence recruitment strategies. This clinical trial tested whether patients with granulomatosis with polyangiitis (GPA) had better outcomes after their GPA was well-controlled if they stayed on a dose of 5 mg/day of prednisone or fully came off prednisone [33]. The online recruitment arm of the study utilized a Patient Advocacy Group (PAG) website and social media platforms (Facebook, Twitter, and Google+) to direct potential participants to a public study website. The study website featured study information, inclusion and exclusion criteria, and requirements for participation.

In addition to helping reduce the time and costs of conducting clinical research studies, novel approaches toward online direct-to-patient recruitment could help ensure clinical research questions are answered in a timelier fashion, ultimately bringing therapeutic advances to greater numbers of individuals. The implementation of such methods could also aide in the evolution of clinical research practices for both rare and common diseases.

The main objective of the current study was to use a reflective, mixed-methods approach, focusing on lessons learned from the TAPIR trial, to design an approach to Web-based, social media recruitment that can be tested across a variety of populations (e.g., rare disease type, age, sex, gender, etc.). This study reports on a comprehensive approach to Web-based, direct-to-patient recruitment.

## Methods

This study aimed to design a means for evaluating whether Web-based and social media platforms can be used effectively to recruit patients with rare disease for clinical research studies. We utilized the consolidated criteria for reporting qualitative research (COREQ) checklist for the framing of this study [34]. This design for Web-based patient recruitment is based on four main methods: (1) an iterative, reflective process to determine which aspects of the TAPIR trial could be used to inform new strategies for development, (2) a comprehensive review of the RDCRN’s portfolio of studies to determine a set of various protocols for testing, (3) in-depth, online patient interviews to determine appropriate theoretical framework for message design based on identified communication preferences, and (4) the establishment of support for all recruitment marketing efforts with Patient Advocacy Groups (PAGs) associated with each population of rare disease involved with the protocols under study.

### How prior research (TAPIR trial) informed current development strategies

Previous implementation research has documented the ways in which an iterative and reflective process aiming to draw lessons from previously published studies aids in the design of interventions and frameworks for subsequent testing [35]. A comprehensive review of findings from the TAPIR trial was conducted to identify problems to be addressed in the current study. This approach relied upon a constant comparative method of examining TAPIR’s features alongside current context to develop feasible approaches to addressing identified problems.

Results of the TAPIR trial included 49 patients in the traditional clinical center recruitment arm, with 10 in the online recruitment arm [33].. Enrollment goals for each arm was 3.3 participants per month, with actual enrollment rates of 0.4 (online recruitment) and 1.8 (traditional recruitment) participants per month. Social media recruitment efforts utilized for the online-recruitment arm resulted in 16,094 individuals visiting the public TAPIR website over an approximate two-year period. Of these website visits, only 82 individuals (0.5%) consented to participate in the trial [27]. Of the 82 individuals that provided consent, only 60 (73%) completed the registration process by answering the questions sent to them via e-mail. Of this, 47 of 60 individuals (78%) were eligible to participate in the study based on their self-reported responses. Such significant drop-off, referred to in digital marketing as a bounce rate, from website clicks to registration completion signifies a problem. High bounce rates typically indicate that a website has not been designed to target the visitors it desires.

Additionally, iterative assessment of these findings indicated that the overall workflow (from recruitment to registration) may have involved too many “clicks” and separate tasks for completion (e.g. registration via e-mail questionnaire) for potential participants. The TAPIR trial website utilized an interactive informed consent form for individuals to enroll in the study. Once a participant completed the IC, they were sent an e-mail to verify their e-mail address with a link to a registration form with further questions about their disease. Alternatively, the traditional recruitment approach occurred through the clinical practices of individual research sites.

The public TAPIR website featured 6 individual pages, with users needing to click at least twice to reach the informed consent page. The interactive informed consent document utilized on the website was a traditional consent form, which can be quite lengthy, with large amounts of information for potential participants to digest. This design goes against the “three click rule” of website design, which suggests that users should be able to find all relevant information in three mouse clicks or fewer to avoid leaving users frustrated [36].

Data also revealed that more than 2/3 patients that did access the website did so via a mobile device; however, the public website’s content was not optimized for mobile use. In the field of health communication, theory is often cited as a crucial component for inclusion in campaign and message development, yet this step was not addressed in the TAPIR study [37]. The evaluation of advertising measures is more organized and cost effective with theoretically-based approaches, as specific measurable constructs can be easily identified and tested [38]. As such, our implementation experience revealed the need for a theoretical construct for message design, complete with message testing phases that incorporate patient feedback.

The TAPIR trial also required online recruitment arm patients to provide their doctors with a Physician packet to complete for confirmation that they were eligible for the trial. Only 35 out of 47 physicians provided additional information about their patients, signifying a loss of patients at this stage, illustrating the importance of seeking ways to engaging clinicians in the recruitment of patients for research [33].

One of the primary lessons learned from the TAPIR study was the need for future research to investigate the ways in which direct-to-patient recruitment, via the online recruitment approach, may differ across various populations. The study only looked at one rare disease population (GPA), which featured mean participant ages of 54.8 years (online recruitment arm) and 55.6 years (traditional recruitment arm). Arguably, this demographic may not be the most appropriate population for consideration of Web-based recruitment techniques, as substantial differences in social media and Internet use by age exist, with only 64% of U.S. adults ages 50–64 using such platforms [39]. As such, it is possible that the online recruitment arm of the study was not as successful because the trial itself, or the population targeted, were not appropriate for consideration across the Web-based landscape.

Although results of the TAPIR study found that the Web-based online recruitment approach was not as effective as the traditional approach, findings did indicate that web-based social media proved successful in mobilizing a substantial number of individuals to the study website [33]. Iterative assessment of the TAPIR study revealed the importance of incorporating existing partnerships with PAGs into the recruitment process, including patients earlier in the recruitment design process, and creating a more detailed, integrated marketing plan that can track the use of social media recruitment tactics. Given that social media recruitment efforts were also reported as appealing via the qualitative data collected from participants, the RDCRN concluded direct-to-patient approach is still highly appealing, with further research needed regarding the implementation of a successful Web-based marketing strategy.

### Protocol selection process

While the TAPIR trial’s online recruitment approach featured a mean patient age of 54.8, the current study comprehensively reviewed all studies being led by the RDCRN to determine which studies would cover the broadest range of patient ages, rare disease categories, and study requirements. The enrolling studies embedded herein address important clinical questions regarding rare diseases. The rare disease research community has identified these topics as important areas of unmet need and these studies have the potential to impact clinical practice. The current study provides the foundation upon which we will test the proposed novel methods for recruitment, data collection, and overall conduct of rare disease clinical research.

A total of six research studies from 5 rare disease consortia (see Table 1) were chosen for inclusion in our efforts to design a comprehensive direct-to-patient recruitment approach, chosen according to those studies which provide a diverse array of study designs and target populations. Factors including research study design (placebo-controlled, observational, longitudinal, etc.), target demographic participant population, type of investigational agent or device used in the study (investigational agent as compared to a repurposed one), and the level of participant involvement in the study (in-person visits, daily diaries, etc.) were considered in the selection of research studies to include in this protocol. Accordingly, there is no planned accrual target overall and the target enrollment will be that of the RDCRN Consortium study accepted as a stratum, that is, there is no change to each study’s target accrual. Each stratum reflects the design of the accepted Consortia protocols.

**Table 1:** RDCRN PRISM Protocols

Protocol	Consortium	Site Locations	Target Accrual	Study Type	Intervention Type	Age	Disease Status
Abatacept (CTLA4-Ig) for the Treatment of Relapsing, Non-Severe, Granulomatosis with Polyangiitis (ABROGATE)	Vasculitis Clinical Research Consortium (VCRC)	US, Canada, UK, Ireland, Germany	66	Interventional RCT, Phase III	• Double-blinded • Placebo-controlled • Investigational agent	15 years old and up	Mild flare- active disease at enrollment
A Randomized, Multicenter Study for Isolated Skin Vasculitis (ARAMIS)	Vasculitis Clinical Research Consortium (VCRC)	US, Canada	90	Interventional sequential multiple assignment RCT	• 3 standard of care medications	18 years old and up	Active disease at enrollment
Longitudinal Evaluation of Autoimmune Pulmonary Alveolar Proteinosis (LongPAP)	Rare Lung Disease Consortium (RLDC)	US	100	Longitudinal, Observational	• None	All ages	Active Disease/ Remission (no major disease activity)
Newer Direct-Acting Anti-Viral Agents as Sole Therapy of Porphyria Cutanea Tarda in Subjects with Chronic Hepatitis C	Porphyrias Consortium (PC)	US	49	Interventional	• Open label • One arm	18 years old and up	PCT with chronic Hepatitis C
A Randomized Double-Blind Controlled Trial of Everolimus in Individuals with PTEN Mutations	Developmental Synaptopathies Consortium (DSC)	US	40	Interventional, Phase I/II	• Placebo-controlled • Investigational agent	5–45 years old	Outpatients with PTEN genetic mutation
A Prospective, Multicenter Study to Compare and Validate Endoscopic, Histologic, Molecular, and Patient-Reported Outcomes in Pediatric and Adult Patients with Eosinophilic Esophagitis, Gastritis, and Colitis	Consortium of Eosinophilic Gastrointestinal Disease Researchers (CEGIR)	US	1050	Observational	• None	3 years old and up	Active disease at enrollment

### Patient interviews

Designing recruitment messages for patient audiences can be challenging, as inclusion and exclusion criteria can seem complex or overly scientific to lay individuals [34]. Therefore, it is important that such messages are designed not only to be attractive, but also to feature content easily understood by audience members [40]. Prior to deciding on messages to be distributed for recruitment, it is essential that formative research be conducted to understand what target audiences want, will attend to, and can understand [41]. Although arguably not often a step taken prior to participant recruitment initiatives, addressing these factors has been shown to increase the probability of behavior change during mass communication campaigns [42]. To facilitate the design of a direct-to-patient recruitment strategy that would best resonate with patients, we implemented in-depth online interviews to test all message iterations created. Institutional Review Board (IRB) approval was obtained prior to any contact with interview and research subjects.

An interview guide (Supplementary File 5) was developed for this study to determine patient preferences for recruitment content platforms, important facets of message design to consider when working with particular rare disease groups, and best times of day to share content via social media. Participants for online interviews were recruited via posts on the RDCRN Facebook page using convenience sampling. Patients recruited were those that represented each of the rare disease categories included in the six chosen protocols. Following the completion of informed consent, patients were individually interviewed via an online video conferencing system (GoToMeeting). All interviews were recorded and lasted 60–90 min. The first author of this study, a female assistant professor whose Ph.D. training focused on qualitative research methods, conducted all online interviews. The lead author took field notes for each interview conducted. A semi-structured approach was used during interviewing, during which participants were shown existing recruitment posts being used by other clinical researchers and asked to provide their feedback. All interview participants received a $10 digital Amazon gift card as compensation for their time.

Between May and September of 2018, 37 individual interviews were conducted with patients across each rare disease category. Overall participant demographics are reflected in Table 2, with supplementary files 1, 2, 3 and 4 providing demographics related to specific disease populations. The interviewer explained to participants that the purpose of the study was to help design appropriate recruitment content, with no interviewer-interviewee relationships established prior to study commencement. Messages evaluated throughout patient concept testing phases included organic social media recruitment content, e-mail blasts to be sent via the RDCRN Contact Registry, and Web design options.

**Table 2:** Overall Patient Characteristics and Demographics

Sex	***n***	%
Female	30	81.1
Male	7	18.9
**Total**	37	100
**Race**	**n**	**%**
American Indian or Alaskan Native	0	0
Asian	0	0
Black or African American	0	0
Native Hawaiian or Pacific Islander	0	0
Caucasian or White	36	97.3
Unknown or Not Reported	1	2.74
**Total**	37	100
**Ethnicity**	**n**	**%**
Hispanic or Latino	0	0
Not Hispanic or Latino	34	91.9
Unknown or Not Reported	3	8.1
**Total**	37	100

**Fig. 1:**
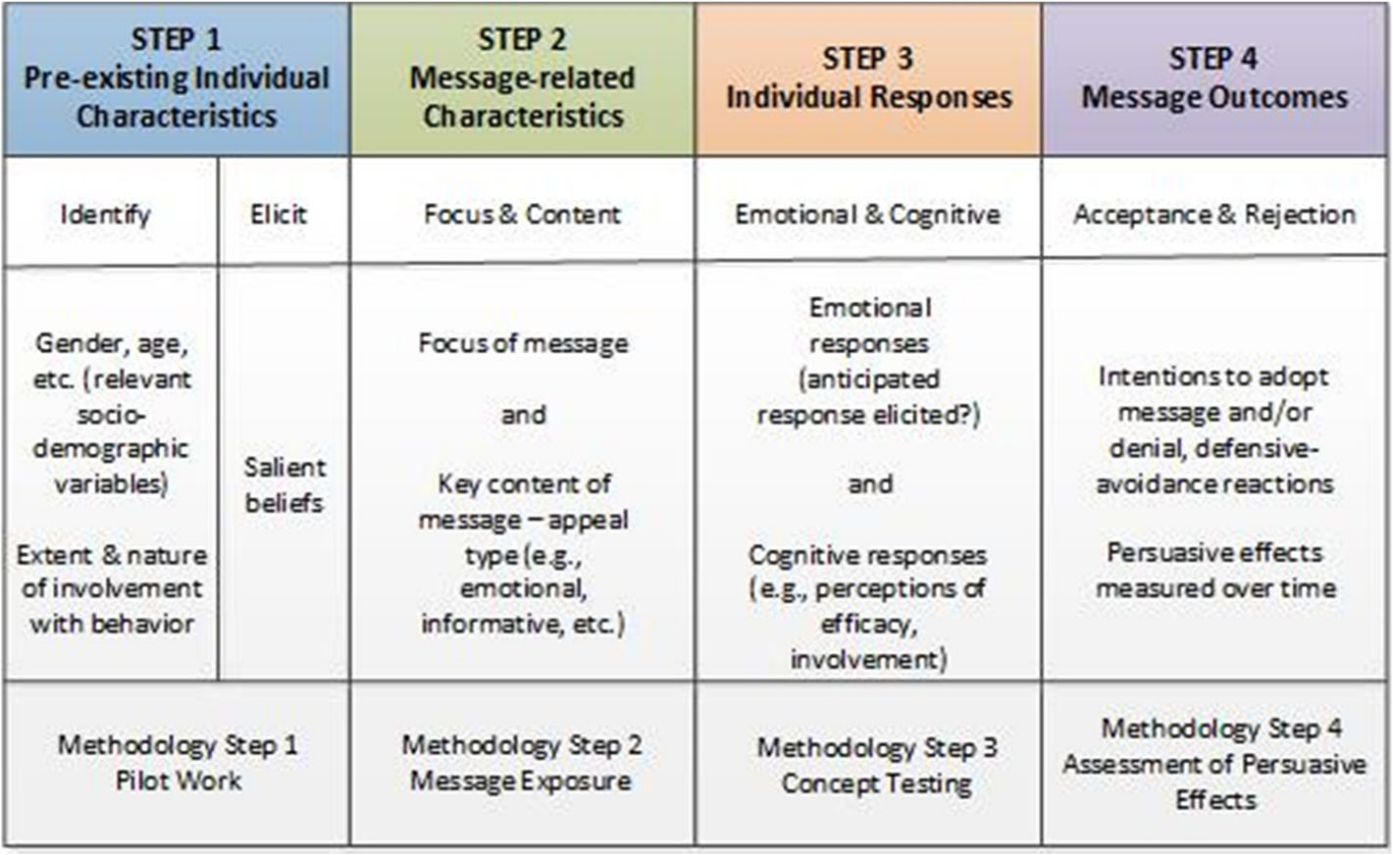
The Step Approach to Message Design and Testing (SatMDT). Source: [37]

All de-identified interview transcripts were coded by first author of this study using NVivo software. In order to enhance the credibility of the data analysis, transcripts were also coded using NVivo’s auto-code feature, which served as a way of triangulating the primary coder’s results. As interviews continued, saturation of responses revealed the points at which our message designs needed to be produced and further edited to align with patient preferences. As such, the Step Approach to Message Design and Testing (SatMDT) was utilized as a theoretical framework to identify target audiences, design message content, pilot test, and evaluate message content (see Fig. 1) for use in the proposed comprehensive approach to direct-to-patient recruitment [43].

### PAG involvement

A principal component of our strategy relied upon soliciting buy-in and commitment from PAG representatives to share and promote our recruitment content via their respective social media platform accounts in order to further promote our efforts. As previously reported by the RDCRN, PAGs act as research partners in ensuring the feasibility and success of various protocols via their collaboration with patient recruitment, support training programs, and study design [2, 44].

PAG representatives were asked to provide what they felt would be the most important information to be considered about their respective target population, including the population’s geographic characteristics, imagery or messaging that should be utilized or avoided, and most successful recruitment strategies to date. These surveys sought to determine a snapshot of each target population to aid the design team in the creation of recruitment messages. Finally, PAGs were asked to provide detailed information regarding their organization’s social media platform preferences, number of followers for each platform, and whether they would be willing to share and promote the RDCRN’s direct-to-patient recruitment content online via their own social media accounts. All PAGs agreed to share this content, with the caveat that all posts be pre-approved by PAG representatives and include a statement reiterating that the promotion of the recruitment content does not mean the PAG is endorsing the particular study being advertised.

## Results

**Fig. 2:**
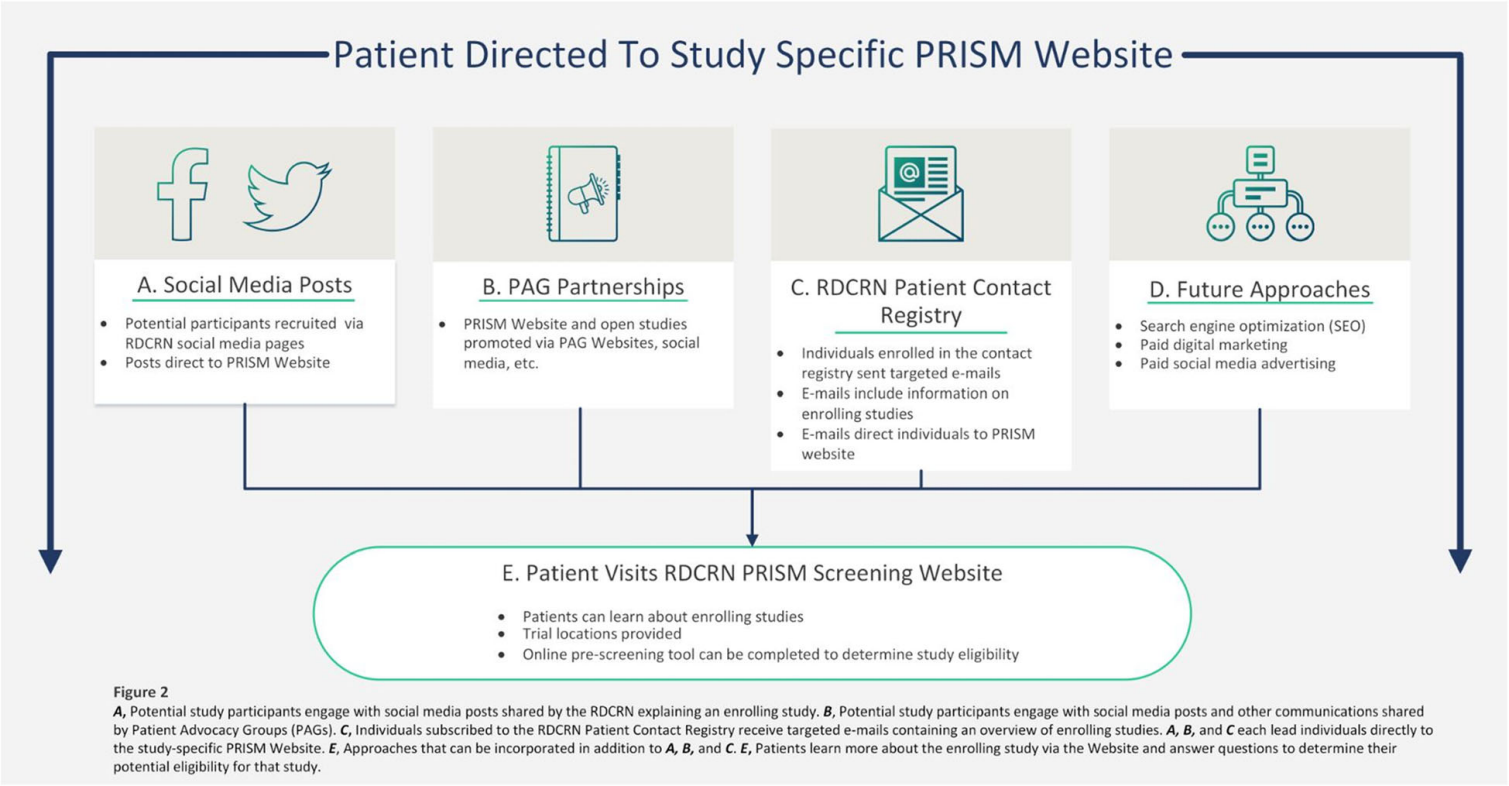
Comprehensive Approach to Identification and Recruitment of Potential Subjects to PRISM Website

Using the previously described methods, we designed an integrated, comprehensive framework for the implementation of social media use in direct-to-patient clinical trial recruitment, titled *Protocol for Increasing accrual using Social Media* (PRISM). PRISM (see Fig. 2) begins with various recruitment efforts (RDCRN social media posts, PAG sharing of RDCRN social media posts, and use of the RDCRN Patient Contact Registry for e-mail blasts) that lead patients to a PRISM public website that acts as a mechanism for patient-initiated screening and subsequent referral.

### How patient feedback and theory informed message design

Consideration of the recruitment messages shared via social media to lead patients to the public PRISM website was an important first-step. In-depth patient interviews utilized existing research recruitment ads and social media posts from other networks (e.g., pharmaceutical companies, non-profit organizations) to help us better understand what kind of content audiences found most appealing. This enabled the creation of more targeted, organic (non-paid) content. Three key message concepts emerged from our analysis of the interview data: (1) infographic; (2) positive emotional messages; and (3) educational information for sharing. Creative designs for Facebook and Twitter were created and iteratively modified throughout each round of message testing. Table 3 describes the message concepts developed based on interview responses.

**Table 3:** PRISM Patient-Preferred Message Concepts

Message Concept	Example Copy	Theme Targeted
Infographic – Study Criteria	You may qualify if: 1) you have been diagnosed with autoimmune PAP^1^, and: 2) are willing to travel to one of our clinical sites 3 times over a 2-year period.	Preference for high-contrast, graphic designs that summarize study eligibility criteria in easy-to-understand terms
Photographed Image – Emotional Appeal	PTEN hamartoma tumor syndrome is a genetic condition in which non-cancerous growths, called hamartomas, develop in different areas of the body. The disease is hereditary, which means it can be passed from parents to their children.	Patients want to feel emotionally connected to images they see featured in the recruitment post; use of family or group images preferred
Educational Post	People affected by PCT^2^ generally experience “photosensitivity,” which causes painful, blistering lesions to develop on sun-exposed areas of the skin (i.e. the hands and face). RDCRN is NOW RECRUITING patients with PCT.	Includes more information about the rare disease that participants can easily share with friends or family to help them understand their diagnosis

^1^Pulmonary alveolar proteinosis

^2^Porphyria cutanea tarda

Based on the feedback obtained from our in-depth interviews with patients, it became clear that social marketing theory emerged as the most useful theoretical framework for informing our message designs. Whereas typical marketing campaigns seek to influence purchasing decisions, social marketing campaigns exist to promote socially desirable behaviors that can help others [45, 46]. Patients often reported that helping to advance science in ways that would help others diagnosed with rare diseases in the future as their main motivator for participating in research. As such, our study designed content focused on the motivator of “helping others” to resonate with target audiences. Examples of our content featuring this theme include tag lines such as “we can’t do this without you” and “your participation helps others with rare diseases.”

Social marketing relies upon a central emphasis on behavior change in coordination with the traditional “4 P’s” of marketing (*product, price, place,* and *promotion*) [47]. Social media and Web-based technologies are being used to elicit the *behavior change* of one choosing to engage with advertisements related to recruitment and ultimately deciding to enroll in a study. In the context of investigators implementing direct-to-patient recruitment via Web-based technologies, the *product* is the PRISM website. The *price* for investigators is minimal, in that the implementation of direct-to-patient recruitment techniques requires time and a marketing plan. *Place* refers to the web-based technologies used to house recruitment materials and the PRISM website. *Promotion* includes the range of integrated advertising and direct-to-patient communication content created that are featured in the results section of this manuscript.

### PRISM public website

Clicking on the Facebook and Twitter posts described above lead participants to a study-specific PRISM public website. Our market research informed each step of the design of these pages. During the interview phase, patients often reported being most likely to engage with a study website that was aesthetically pleasing, easy to navigate, and included pertinent study information written in a way that they could understand. As these three themes were the primary findings of our interviews, we began designing mock website templates that were shared with patients as interviews continued. We engaged in a constant iterative process where interview feedback was incorporated to edit the website. Once saturation was reached with a Website mockup that ongoing interviews found was well received by patients, the final base Website was built.

**Fig. 3:**
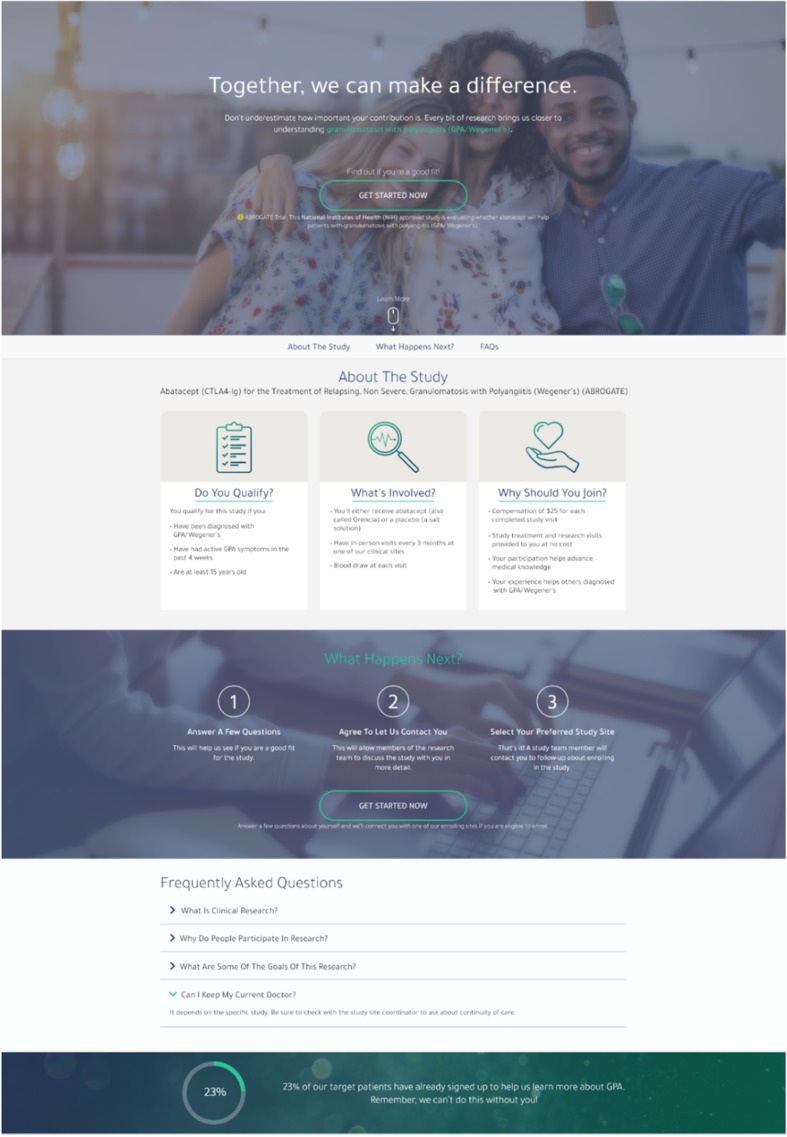
PRISM Website

The structure and aesthetic of the base study website was designed to be easily adaptable and customizable for specific studies. The PRISM website provides easy-to-understand, summarized information for patients, such as inclusion/exclusion criteria, study design, and how to participate. Patients felt strongly that the color scheme of the website needed to be professional, yet bright, and they often emphasized their support for the use of the background image (a group of people) because this made them feel they were working together toward a common goal of helping others (see Fig. 3).

**Fig. 4:**
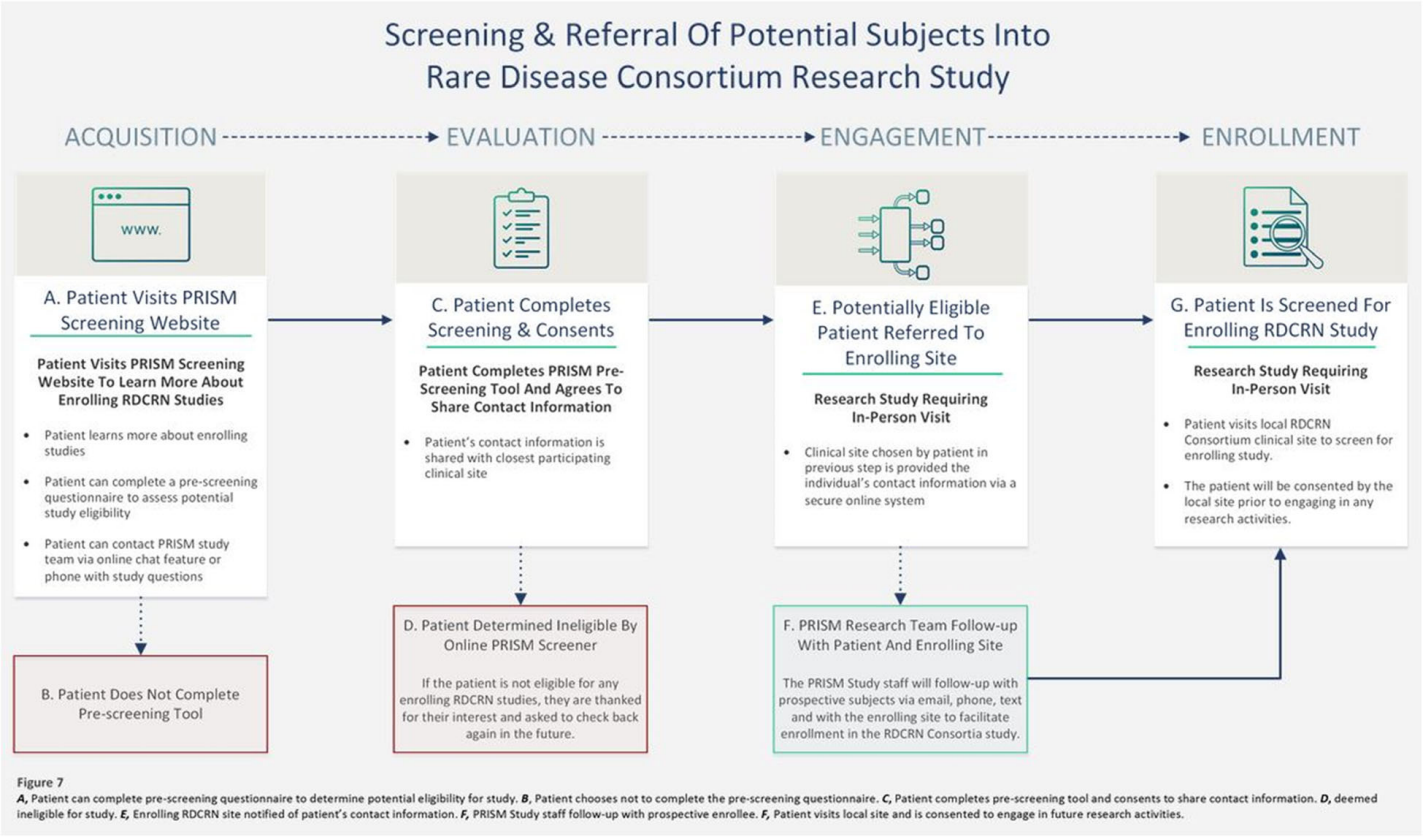
Screening & Referral of Potential Subjects into Rare Disease Consortium Research Study. Figure generated using Microsoft Visio

**Fig. 5:**
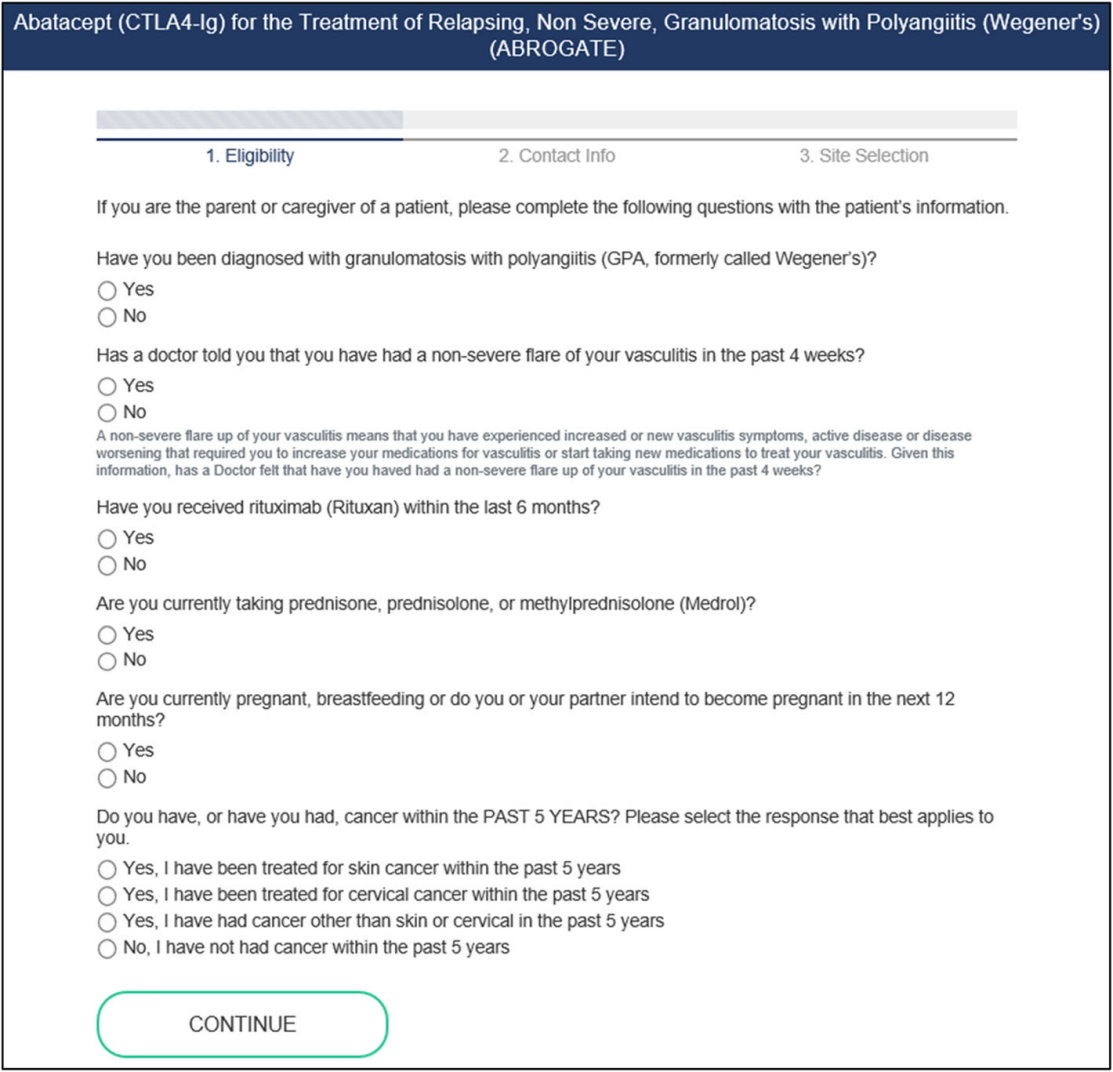
Online Screener Example

The Website workflow (see Fig. 4) begins with patients visiting the Website, where they can learn more information about the study. Individuals are then given the opportunity to screen for their potential eligibility by completing an interactive questionnaire with ten items or less (see Fig. 5). All screening questions were developed based on appropriate health literacy criteria (e.g., using simple language, defining technical terms, using active voice in messaging) and in conjunction with study Principal Investigators (PIs) [48]. If the participant is found to be eligible for a recruiting Consortium clinical study based on self-reported responses, the participant will move to the registration phase. Here, the patient will provide their contact information and agree to share this information with PRISM staff and with the enrolling clinical center in their geographic area for studies requiring in-person studies. Patients may also indicate their contact preferences in this step to indicate how study staff may contact them (via phone, email, or text).

For RDCRN Consortia studies that require in-person visits, patients that are deemed potentially eligible based on their self-reported screening responses on the PRISM website will have the option to consent to share their contact information with the RDCRN clinical site of their choice. The PRISM registration process will assist patients in finding the closest enrolling site geographically to them using an embedded Google Maps feature. Each of the rare disease studies chosen for PRISM were selected based on having a varying number of clinical sites and differences in geographic location of sites, allowing us to examine participant willingness to travel for rare disease research once PRISM launches to the public. Based on anecdotal evidence provided by investigators and PAGs, many rare disease patients are willing to travel for their healthcare needs. Geographic availability of site locations for patients is one of the variables our study team plans to explore on acceptance and enrollment of patients in the trial and the subseuqnet response to the PRISM initiative once it is launched. We are interested to examine is a patient’s willingness to travel may vary based on features related to the disease population and/or the study intervention. PRISM will further allow us to effectively analyze this conjecture based on the type of study and target disease population.

Patients that do not have a site geographically located near them will have a tool on the interactive website to note, “I do not see a referring site near me” if no enrolling sites are close to their geographic location. These subjects will be referred to the sponsor’s study team for follow-up to determine if the patient is able to travel to a participating site or participate remotely, if permitted by the study protocol.

PRISM is a potential participant identification and referral tool. The participant is under the DMCC’s IRB for PRISM recruitment and the online pre-screening questionnaire, and must consent to share their contact information with the enrolling RDCRN clinical site of their choice. Once patients have agreed to share their contact information, the patient’s contact information and self-reported responses will be provided to the enrolling clinical site via a secure password-protected online members’ website maintained by the DMCC. The enrolling clinical site staff will receive an automated notification email alerting site staff that a new patient was referred to their site.

The enrolling clinical site will then contact the referred participant to further assess eligibility for the research study and attempt to bring the participant in for an in-person screening or electronic visit (for studies that do not require in-person visits) if deemed eligible. Once the patient has been referred to the enrolling RDCRN Consortium clinical site, the patient will be under the purview of the local site’s IRB approved protocol for enrollment in the research study. Thus, the patient must be consented and screened for enrollment in the study as any traditionally recruited subject (for in-person or electronic studies).

For example, a potential study participant with GPA completes the online PRISM screener for the VCRC 5527 Abatacept (CTLA4-Ig) for the Treatment of Relapsing, Non-Severe, Granulomatosis with Polyangiitis (ABROGATE) trial. The online PRISM recruitment via social media, PRISM website self-pre-screening questionnaire and online informed consent form to share their contact information fall under the DMCC’s IRB for PRISM-related activities. Once the patient is referred to an enrolling RDCRN clinical site, the local site will need to use their IRB-approved informed consent form for the ABROGATE trial, further discuss the study with the individual, and consent them for the study. The PRISM IRB protocol only covers the recruitment, online pre-screening, and referral to enrolling RDCRN clinical sites. Once referred, the patient will follow the local enrolling clinical site’s IRB approved enrollment procedures (for both in-person and electronic studies). For studies that do not require in-person visits, the patient would still fall under the study’s IRB.

Clinical site personnel will document whether the patients referred to their site via PRISM were deemed eligible by the clinical site staff and if the PRISM participant later enrolls in the clinical study. Enrollment metrics will be regularly evaluated to determine if the online recruitment and referral process requires modifications to improve the screening and referral of potentially eligible research study participants.

Throughout the screening and referral process bidirectional communications via phone, email and text between PRISM study staff and potential subjects will be available to assist with any technical questions the patient may have regarding the online screener and to facilitate enrollment in the RDCRN study. PRISM study staff may also communicate with prospective enrollees and clinical sites throughout this process to ensure the PRISM participant was contacted by the clinical site staff and assist in triaging any questions or concerns the prospective enrollee may have regarding the study enrollment process.

The funding for ongoing PRISM study staff communication with potential study participants was under the main NIH award for the DMCC at USF. As part of the PRISM initiative, we are evaluating the amount of resources required for participant follow-up (number of e-mails, phone calls, etc.) and working to automate as many processes as possible to reduce personnel demand. Evaluating the return on investment for telephone or manual follow-up of referred subjects is an important component of the PRISM project to determine efficacy and vitality of the initiative moving forward.

Participant satisfaction with the enrollment process will be solicited following enrollment and regularly monitored by PRISM staff to evaluate and improve the recruitment and referral process. Feedback from RDCRN Consortia clinical site staff and Consortia investigators will be welcomed and incorporated to enhance the screening and referral processes for PRISM.

Data collected for RDCRN study participants identified and enrolled via the PRISM model will be compared to RDCRN study participants recruited and enrolled through other channels to determine if the participant populations recruited through these different avenues differ in demographics, compliance, withdrawal rates or other study metrics.

## Discussion

Evidence suggests that as many as 19% of clinical trials close without meeting at least 85% of target accrual rates, signifying the necessity to investigate new methods for implementing novel approaches to research recruitment [49]. This study lead to the design of a comprehensive direct-to-patient recruitment plan to assist in promoting patient opportunities for research, to achieve the goals of the research to improve the health of individuals [50]. Based on lessons learned from the TAPIR trial, PRISM was designed to provide a simple, streamlined process for patients with rare diseases to self-identify and discover clinical research studies for which they may be eligible to enroll.

PRISM is an online recruitment approach that heavily utilized patient feedback in the production, design, and editing of all recruitment posts and the public Website. The process of comparing and contrasting different protocol types to develop one’s own “best practices” for recruitment of specific populations is a logical step for maximizing recruitment efforts. We focused on the rare disease population, but it is clear that there is not a “one size fits all” approach to direct-to-patient recruitment efforts. It is important that any approach to message design taken be done in accordance with market segmentation. The concept of market segmentation (pre-existing individual characteristics) focuses on categories such as gender, average age, and other aspects relating with an identified group when tailoring messages for dissemination [51]. By clearly defining target audiences (e.g., clusters of particular individuals within each rare disease category), researchers can better define message strategies to enhance the likelihood patients will be influenced by the messaging [18, 19, 52].

To design an appropriate means for evaluating the efficacy of Web-based recruitment strategies, it was considered important that various study populations and trial designs be chosen for inclusion in the new project. Broadening the scope of patient populations allows for the comparison of recruitment strategies based on: the characteristics of the RDCRN study design (e.g. randomized trial, longitudinal study, etc.), demographics of the study population of interest, study requirements of the participant (e.g. online survey, in-person clinical trial, etc.) through metrics such as rate of recruitment, percent eligible, percent drop-out after consent, time from first contact to enrollment, and qualitative feedback from referred patients and RDCRN study staff.

The PRISM website is innovative in that its design and content are patient-focused. Other online research study finder websites (e.g., ClinicalTrials.gov) pull study inclusion and exclusion criteria directly from study protocols, which can be quite confusing for patients to self-navigate without assistance from healthcare personnel. This process also differs from other online research study finders in that the PRISM provides a communication channel so patients can ask questions to study staff in real time and allow for a personalized study staff member to follow-up with patients to assist with enrollment. This customized enrollment experience seeks to personalize the referral process with potential subjects and assist with eliminating barriers to enrollment in research studies.

The public website designed and used for the TAPIR study included 6 pages, with many user clicks required to reach the informed consent page. The informed consent page itself was lengthy, with qualitative data revealing that patients were often left confused by the questions posed. In response to this, PRISM was designed as a more organized, streamlined process, with primary study information and a link to screen available on the main page and screening questions available on a second page. PRISM adjusted the order in which patients could screen for the study, with screening questions made available to patients before requesting consent to share their contact information. Patients are only asked to consent to share this information if they are deemed to be eligible for the study. In addition, no more than 10 questions are included for patient screening in an effort to keep the process manageable and efficient for patients. Patient information is collected at one time with PRISM, providing an opportunity for clinical sites to more quickly follow-up with patients about enrolling in their respective studies.

In response to the significant use of mobile devices for accessing the TAPIR study information, PRISM was optimized to be mobile-friendly, with visual icons incorporated to make it easier for patients to read and understand the content being presented via their mobile devices.

In the TAPIR study, patients that did consent tended to drop-off (did not enroll in the study) following their completion of the informed consent form. Part of this may have been because patients were required to verify their e-mail address prior to completing their registration. PRISM, therefore, incorporates a more focused workflow, where patients can read more about the study, screen, and consent to share their contact information all in one step.

Finally, in the TAPIR trial, patients provided their physicians with a packet to complete to confirm their eligibility for the study. Only 74% of physicians provided this information. Therefore, PRISM removes this barrier. Rather than giving patients the task of having this packet completed, patients are now connected directly with enrolling sites after consenting to provide their contact information.

### Limitations

As in most studies that collect qualitative data, it is difficult to generalize the responses of our rare disease patient population to larger audiences. Individuals with rare diseases may arguably be more enthusiastic and engaged about finding research opportunities, as this population has fewer options in this area when compared to other, more medically-recognized diseases or diagnoses. However, the current study provides important areas for consideration for any area of medicine in the development of approaches to direct-to-patient recruitment. Additionally, exclusively using a Website to find potential participants may preclude individuals who do not have Internet access or use social media from being included in the discovery of study opportunities. As such, it is important that general practitioners and medical staff also advertise and promote the Website’s content in order to include all potential participants.

## Conclusions

This study presents one of the first formal applications of previous data to inform the theory based creation of a comprehensive approach to conducting Web-based direct-to-patient recruitment for research. The development of PRISM builds upon the positive findings in the TAPIR trial, seeks to avoid the challenges identified in the approach used for TAPIR, and advances innovative approaches to clinical research by: (1) expanding the use of social media and other online recruitment strategies through the use of novel technologies and marketing campaigns to target populations of patients with rare diseases previously unavailable to researchers [20–24]; (2) identifying and attempting to remove barriers to enrollment and participation in clinical research studies through web-based referral [53]; and (3) demonstrating the feasibility of the proposed methods for clinical research studies in rare disease populations.

## Supplementary information

**Additional file 1.** Supplementary File 1. Interview Demographics – Participant Disease Type. Interview demographics by disease type

**Additional file 2.** Supplementary File 2. Interview Demographics – Participant Sex. Interview demographics by sex

**Additional file 3.** Supplementary File 3 Interview Demographics – Participant Race. Description of data: Interview demographics by race

**Additional file 4.** Supplementary File 4. Interview Demographics – Participant Ethnicity. Interview demographics by ethnicity

**Additional file 5.** Supplementary File 5 PRISM Patient Participant Interview Guide

## Data Availability

The datasets used and/or analyzed during the current study are available from the corresponding author on reasonable request.

## Abbreviations

ABROGATE: Abatacept (CTLA4-Ig) for the Treatment of Relapsing, Non-Severe, Granulomatosis with Polyangiitis
CI: Consortium Investigator
DMCC: Data Management and Coordinating Center
FDA: Food and Drug Administration
GPA: Granulomatosis with polyangiitis
IRB: Institutional Review Board
NCATS: National Center for Advancing Translational Sciences
ORDR: Office of Rare Diseases Research
PAP: Pulmonary Alveolar Proteinosis
PCR: Patient Contact Registry
PCT: Porphyria Cutanea Tarda
PI: Principal Investigator
PM: Project Manager
PRISM: Protocol for Increasing accrual using Social Media
PTEN: Phosphatase and Tensin Homolog
RCT: Randomized Controlled Trial
RDCRN: Rare Diseases Clinical Research Network
TAPIR: The Assessment of Prednisone in Remission Trial
UK: United Kingdom
US: United States
USF: University of South Florida
