# Supplementary material for: A novel approach to conducting clinical trials in the community setting: utilizing patient-driven platforms and social media to drive web-based patient recruitment
Source: BMC Med Res Methodol. 2020 Mar 13;20:58. doi: 10.1186/s12874-020-00926-y (PMC7069058; doi:10.1186/s12874-020-00926-y)
Supplement: Supplementary file 1 — Additional file 1. Supplementary File 1. Interview Demographics – Participant Disease Type. Interview demographics by disease type [file 12874_2020_926_MOESM1_ESM.docx]

**Supplementary File 1**

EoE: Eosinophilic esophagitis

EG: Eosinophilic gastritis

EC: Eosinophilic colitis

PCT: Porphyria cutanea tarda

PAP: Pulmonary alveolar proteinosis

GPA: Granulomatosis with polyangiitis

IgA Vasculitis: formerly known as Henoch-Schönlein purpura
