# Supplementary material for: A novel approach to conducting clinical trials in the community setting: utilizing patient-driven platforms and social media to drive web-based patient recruitment
Source: BMC Med Res Methodol. 2020 Mar 13;20:58. doi: 10.1186/s12874-020-00926-y (PMC7069058; doi:10.1186/s12874-020-00926-y)
Supplement: Supplementary file 2 — Additional file 2. Supplementary File 2. Interview Demographics – Participant Sex. Interview demographics by sex [file 12874_2020_926_MOESM2_ESM.docx]

**Supplementary File 2**

EoE: Eosinophilic esophagitis

EG: Eosinophilic gastritis

EC: Eosinophilic colitis

PCT: Porphyria cutanea tarda

PAP: Pulmonary alveolar proteinosis

GPA: Granulomatosis with polyangiitis

IgA Vasculitis: formerly known as Henoch-Schönlein purpura
