# Supplementary material for: A novel approach to conducting clinical trials in the community setting: utilizing patient-driven platforms and social media to drive web-based patient recruitment
Source: BMC Med Res Methodol. 2020 Mar 13;20:58. doi: 10.1186/s12874-020-00926-y (PMC7069058; doi:10.1186/s12874-020-00926-y)
Supplement: Supplementary file 3 — Additional file 3. Supplementary File 3 Interview Demographics – Participant Race. Description of data: Interview demographics by race [file 12874_2020_926_MOESM3_ESM.docx]

**Supplementary File 3: Interview Demographics – Participant Race**

|  | **Race** | | | | | | | | | | | | |
| --- | --- | --- | --- | --- | --- | --- | --- | --- | --- | --- | --- | --- | --- |
|  | **American^[[1]](#footnote-1)^** | | **Asian** | | **Black^[[2]](#footnote-2)^** | | **Pacific Islander^[[3]](#footnote-3)^** | | **White** | | **Unknown^[[4]](#footnote-4)^** | | |
| **Disease** | ***n*** | **%** | ***n*** | **%** | ***n*** | **%** | ***n*** | **%** | ***n*** | **%** | ***n*** | **%** |  |
| CEGIR 7801: EoE^[[5]](#footnote-5)^, EG^[[6]](#footnote-6)^, EC^[[7]](#footnote-7)^ | 0 | 0 | 0 | 0 | 0 | 0 | 0 | 0.0 | 6 | 100 | 0 | 0 |  |
| DSC 7904: Cowden syndrome | 0 | 0 | 0 | 0 | 0 | 0 | 0 | 0 | 1 | 100 | 0 | 0 |  |
| PC 7210: PCT^[[8]](#footnote-8)^ | 0 | 0 | 0 | 0 | 0 | 0 | 0 | 0 | 0 | 0 | 0 | 0 |  |
| RLD 5712: PAP^[[9]](#footnote-9)^ | 0 | 0 | 0 | 0 | 0 | 0 | 0 | 0 | 5 | 100 | 0 | 0 |  |
| VCRC 5527: GPA^[[10]](#footnote-10)^ | 0 | 0 | 0 | 0 | 0 | 0 | 0 | 0 | 20 | 100 | 0 | 0 |  |
| VCRC 5562: Skin/Cutaneous vasculitis, IgA Vasculitis^[[11]](#footnote-11)^ | 0 | 0 | 0 | 0 | 0 | 0 | 0 | 0 | 4 | 80 | 1 | 20 |  |
| ***Total*** | *0* | *0* | *0* | *0* | *0* | *0* | *0* | *0* | *36* | *97.3* | *1* | *2.74* |  |

1. Including Indian, Alaskan, and Native [↑](#footnote-ref-1)
2. Including African and/or American [↑](#footnote-ref-2)
3. Including native Hawaiian [↑](#footnote-ref-3)
4. Including not reported [↑](#footnote-ref-4)
5. Eosinophilic esophagitis [↑](#footnote-ref-5)
6. Eosinophilic gastritis [↑](#footnote-ref-6)
7. Eosinophilic colitis [↑](#footnote-ref-7)
8. Porphyria cutanea tarda [↑](#footnote-ref-8)
9. Pulmonary alveolar proteinosis [↑](#footnote-ref-9)
10. Granulomatosis with polyangiitis [↑](#footnote-ref-10)
11. formerly known as Henoch-Schönlein purpura [↑](#footnote-ref-11)
