# Supplementary material for: A novel approach to conducting clinical trials in the community setting: utilizing patient-driven platforms and social media to drive web-based patient recruitment
Source: BMC Med Res Methodol. 2020 Mar 13;20:58. doi: 10.1186/s12874-020-00926-y (PMC7069058; doi:10.1186/s12874-020-00926-y)
Supplement: Supplementary file 4 — Additional file 4. Supplementary File 4. Interview Demographics – Participant Ethnicity. Interview demographics by ethnicity [file 12874_2020_926_MOESM4_ESM.docx]

**Supplementary File 4: Interview Demographics – Participant Ethnicity**

|  | **Ethnicity** | | | | | |
| --- | --- | --- | --- | --- | --- | --- |
|  | **Hispanic or Latino** | | **Not Hispanic or Latino** | | **Missing^[[1]](#footnote-1)^** | |
| **Disease** | ***n*** | **%** | ***n*** | **%** | ***n*** | **%** |
| CEGIR 7801: EoE^[[2]](#footnote-2)^, EG^[[3]](#footnote-3)^, EC^[[4]](#footnote-4)^ | 0 | 0.0 | 6 | 100 | 0 | 0.0 |
| DSC 7904: Cowden syndrome | 0 | 0.0 | 1 | 100 | 0 | 0.0 |
| PC 7210: PCT^[[5]](#footnote-5)^ | 0 | 0.0 | 0 | 0.0 | 0 | 0.0 |
| RLD 5712: PAP^[[6]](#footnote-6)^ | 0 | 0.0 | 5 | 100 | 0 | 0.0 |
| VCRC 5527: GPA^[[7]](#footnote-7)^ | 0 | 0.0 | 20 | 100 | 0 | 0.0 |
| VCRC 5562: Skin/Cutaneous vasculitis, IgA Vasculitis^[[8]](#footnote-8)^ | 0 | 0.0 | 2 | 40.0 | 3 | 60.0 |
| ***Total*** | *0* | *0.0* | *34* | *91.9* | *3* | *8.1* |

1. Including patients who refused to participate [↑](#footnote-ref-1)
2. Eosinophilic esophagitis [↑](#footnote-ref-2)
3. Eosinophilic gastritis [↑](#footnote-ref-3)
4. Eosinophilic colitis [↑](#footnote-ref-4)
5. Porphyria cutanea tarda [↑](#footnote-ref-5)
6. Pulmonary alveolar proteinosis [↑](#footnote-ref-6)
7. Granulomatosis with polyangiitis [↑](#footnote-ref-7)
8. formerly known as Henoch-Schönlein purpura [↑](#footnote-ref-8)
