# Supplementary material for: A novel approach to conducting clinical trials in the community setting: utilizing patient-driven platforms and social media to drive web-based patient recruitment
Source: BMC Med Res Methodol. 2020 Mar 13;20:58. doi: 10.1186/s12874-020-00926-y (PMC7069058; doi:10.1186/s12874-020-00926-y)
Supplement: Supplementary file 5 — Additional file 5. Supplementary File 5 PRISM Patient Participant Interview Guide [file 12874_2020_926_MOESM5_ESM.docx]

**Supplementary File 5**

**PRISM Patient Participant Interview Guide**

*(Goals: learn about patient’s experience with a rare disease, gauge patient feelings on incentives and barriers to participating in medical research, and obtain feedback on current message design – in what ways is our current approach working? What can we edit in our messaging to reflect a more patient-centric theme?)

The following questions are designed to be semi-structured in nature, meaning that the interviewer will be able to engage in a conversation with the participant. Follow-up questions related to the above goals will be asked when appropriate.*

Semi-Structured Interview Questions

1. To begin, I’d like to ask you to tell me more about your rare disease. Explain to me your diagnosis and what your experience has been like for you.
   1. When were you diagnosed?
   2. Can you describe to me what it feels like to have that type of rare disease?
   3. Think back to your time of diagnosis. If you used the Internet (e.g. Google) to search for information about your diagnosis, what search terms did you enter?
2. Tell me the first thoughts that pop into your head when I say “research study…” “clinical trial….” “medical study…”
   1. Have you ever participated in a research study that investigated your rare disease?
   2. If yes, please describe what it felt like to be a participant in that study.
   3. If yes, what did you/did you not like about being a participant in a research study?
   4. If yes, how comfortable were you with the idea of participating in a medical research study?
   5. If no, would you ever consider participating in a medical research study or clinical trial? Why or why not?
   6. If no, can you please explain why you have not participated in such a study thus far?

3. Do you use social media in relation to your rare disease? How so? Why or why not?

 a. Which social media platforms do you use? Why? How often do you use these
 platforms?

b. If you are a member of certain groups, please name the groups and their
 platforms.

c. Have you switched at all in using one social media platform related to your rare
 disease versus another? If so, why?

d. Thoughts on Twitter? Pinterest? Instagram? Reddit? SnapChat? In relation to
 your rare disease?

e. What about other sites that you may not consider social media? Health Unlocked?
 Patients Like Me?

4. I have been working with some of my colleagues to pull other messages being used on
 social media in attempting to recruit patients like you for clinical trials. The purpose of
 these messages are to help increase recruitment for clinical trials or research studies. If it
 is okay with you, I’d like to show you a few of the messages and get your feedback.
 [show social media content/posts]

a. Are these messages relevant to you? Why or why not?

b. What did you learn from the messages? What do you remember the most?

c. Can you describe how you feel when you look at this message?

d. Is there a particular part of this message that you like or dislike the most?

e. Do you feel that this message was designed with you in mind? Why or why not?

f. What would you change about the message?

- 1. Would it be useful if we included summaries of the latest research findings as related to your rare disease?
  2. Would it be useful if we shared personal stories from other patients and their families?
  3. Would it be useful if we included video or audio features in our social media postings?

5. I have also been working with some of my colleagues to develop websites for individual research studies to be shown to patients like you. The purpose of the website I am about to share with you is to help inform you of a research study you may be eligible for. I’d like to encourage you to talk aloud as you look through the website – feel free to tell me what you are thinking or feeling as you are exploring. Then I’ll ask you a series of questions. [show website mock-ups]

a. Is this website relevant to you? Why or why not?

b. What are your opinions on the aesthetic design of the website (look/feel/colors)?

d. Do you feel that this website was designed with you in mind? Why or why not?

e. What would you change about the website?

f. Does this website motivate you to participate in the study? Why or why not?
